# *GIGANTEA* Unveiled: Exploring Its Diverse Roles and Mechanisms

**DOI:** 10.3390/genes15010094

**Published:** 2024-01-13

**Authors:** Ling Liu, Yuxin Xie, Baba Salifu Yahaya, Fengkai Wu

**Affiliations:** 1 Faculty of Agriculture, Forestry and Food Engineering, Yibin University, Yibin 644000, China; lingliu112@163.com; 2 Maize Research Institute, Sichuan Agricultural University, Chengdu 611130, China; xieyx4301@163.com (Y.X.); bsyahayat@yahoo.com (B.S.Y.); 3 Key Laboratory of Biology and Genetic Improvement of Maize in Southwest Region, Ministry of Agriculture, Chengdu 611130, China

**Keywords:** *GI*, circadian clock, flowering time, stress tolerance, stimulus response

## Abstract

GIGANTEA (GI) is a conserved nuclear protein crucial for orchestrating the clock-associated feedback loop in the circadian system by integrating light input, modulating gating mechanisms, and regulating circadian clock resetting. It serves as a core component which transmits blue light signals for circadian rhythm resetting and overseeing floral initiation. Beyond circadian functions, *GI* influences various aspects of plant development (chlorophyll accumulation, hypocotyl elongation, stomatal opening, and anthocyanin metabolism). *GI* has also been implicated to play a pivotal role in response to stresses such as freezing, thermomorphogenic stresses, salinity, drought, and osmotic stresses. Positioned at the hub of complex genetic networks, *GI* interacts with hormonal signaling pathways like abscisic acid (ABA), gibberellin (GA), salicylic acid (SA), and brassinosteroids (BRs) at multiple regulatory levels. This intricate interplay enables *GI* to balance stress responses, promoting growth and flowering, and optimize plant productivity. This review delves into the multifaceted roles of *GI*, supported by genetic and molecular evidence, and recent insights into the dynamic interplay between flowering and stress responses, which enhance plants’ adaptability to environmental challenges.

## 1. Introduction

Agricultural productivity is limited by multiple abiotic stresses, which affects plant growth and development. These stresses induce intricate alterations in cellular metabolism, necessitating adjustments in the central metabolic network of plants to maintain cellular and metabolic homeostasis [1]. The limiting effect of these stresses on crops is intensified by climate change, emphasizing the need for cultivars with enhanced adaptability to ensure global food security [2,3,4]. Plants have evolved intricate mechanisms to cope with abiotic stresses, including regulatory pathways involved in stress signal perception, transduction, transcriptional regulation, and protein modifications [5,6]. Functional genomic approaches, such as high-throughput transcriptomics and proteomics, have contributed to identifying stress-responsive genes and proteins, which have enhanced our understanding of plant responses to environmental challenges [7,8].

Stress-responsive genes fall into two functional categories, playing crucial roles in plant adaptation to abiotic stress [9]. The first category comprises regulatory proteins, including transcription factors, protein kinases, phosphatases, and calcium receptors. These regulators participate in signal transduction pathways by influencing the expression of downstream stress-inducible genes. The second category encompasses diverse protein molecules, such as water channel proteins, chaperones, sugar and proline transporters, osmotin, detoxification enzymes, anti-freezing proteins, and late embryogenesis abundant (LEA) proteins [10,11,12,13]. These proteins act together and contribute to the multifaceted responses employed by plants to mitigate the adverse effects of abiotic stress.

The phenotype of *GI* was initially identified as a late-flowering mutant (*gi*) in *Arabidopsis thaliana* [14,15]. The dynamic responsiveness of *GI* across various developmental stages underscores its active involvement in physiological processes such as seed dormancy, germination, hypocotyl emergence, circadian clock regulation, flower initiation, carbohydrate metabolism, and stress responses [16,17,18,19,20,21]. The intricate temporal regulation of *GI* during diurnal cycles highlights its interconnection with the circadian clock [22], showcasing its pivotal role in coordinating plant temporal responses. *GI*’s influence extends from breaking seed dormancy to circadian clock regulation and stress responses. Its significant role in carbohydrate metabolism underscores its versatility in regulating adaption to environmental challenges [19,20,21]. The regulation and stability of GI’s protein are paramount to normal functioning of the circadian clock system [23,24]. Transcriptomic studies reveal *GI*’s widespread impact on nearly 80% of all genes in plants species like rice (*Oryza sativa*), poplar (*Populus trichocarpa*), and Arabidopsis (*Arabidopsis thaliana*), emphasizing its central position in temporal coordination in plants [25].

## 2. Structural Insights into *GI*

### 2.1. Structural Conservation and Functional Dynamics

*GI* encodes a protein comprising 1173 amino acids with a unique biochemical profile and no homology to any characterized proteins in land plants [18,26]. Despite lacking conserved protein domains [23,27], recent discoveries of *GI* homologues in charophytes like *Coleochaete irregularis* and *Cylindrocystis cushleckae* have expanded our understanding of the GI protein and its functions [28]. The emergence of *GI* in terrestrial plants is observed predominantly as single copies in species like *Arabidopsis thaliana*, *Oryza sativa*, and *Selaginella moelenendorffii*, highlighting its key role in early plant evolution [29]. Interestingly, *GI* is absent in *Physcomitrella patens*, a moss species, but present in charophytes and liverworts like *Marchantia polymorpha* and *Amynthas agrestis*, signifying its unique evolutionary pattern [30]. Further exploration through BLAST searches in OneKP databases emphasized the rarity of *GI* homologues, with the early divergent moss *Takakia lepidozioidea* standing as the sole representative among 41 moss species [28]. This emphasizes the unique evolutionary trajectory of *GI*, indicating its origin in charophytes, the presumed sister lineage to land plants, and its subsequent loss within the moss lineage. In *Solanaceae*, a notable exception, *GI* is found in two or three copies, introducing variability not observed in other plant families [31]. Phylogenetic analysis further delineates separate clades for *GI* in Petunia and *Solanaceae* compared to *Brassicaceae*, *Rosaceae*, and *Fabaceae* [32]. 

Wild-type (WT) alleles play crucial roles in encoding gene products that are required for specific biological functions. Mutations in these WT alleles can lead to loss of the gene functions they encode. The *GI* gene, in particular, has been extensively studied through various mutant alleles, revealing a pleiotropic phenotype with significant effects on multiple aspects of plant biology, including flowering, photoperiodic response, phytochrome B signaling, circadian clock regulation, and carbohydrate metabolism [18,19]. The initial investigation into *GI* involved the study of *gi* mutants, which were characterized by a late-flowering phenotype [33]. Subsequent studies identified several *gi* mutant alleles, such as *gi-1*, *gi-2*, *gi-3*, *gi-4*, *gi-5*, *gi-6*, *gi-11*, *gi-12*, *gi-100*, *gi-200*, *gi-201*, *gi-596*, and *gi-611*, each of which influence distinct biological processes based on the specific location of the mutation in the GI protein’s genome [23]. 

Under long-day (LD) conditions, most *gi* mutant alleles display late-flowering phenotypes, in sharp contrast to mutants of timing of cab expression-1 (*toc1-1*) or late elongated hypocotyl 11 (*lhy-11*), which cause short period rhythms and promote flowering under short-day (SD) conditions [34]. Noteworthy findings include reduction in the susceptibility of *gi*-100 mutants to *Fusarium oxysporum* infection compared to WT plants [17]. Mutant alleles *gi-1* and *gi-2* altered the duration of circadian rhythms in leaf movement, with *gi-1* affecting the expression of *chlorophyll a*/*b binding protein* (*CAB*) and *gi-2* prolonging its expression [18]. Chlorophyll fluorescence (Fv/Fm) was higher in *gi-201* and *gi-2* mutants, suggesting that *GI* could function as a negative regulator of chlorophyll biosynthesis [35]. Certain *gi* mutants displayed impaired phytochrome B signaling and elongated hypocotyls under red light signaling [19]. Moreover, *gi-1* mutants were found to be hypersensitive to drought stress due to increased stomatal aperture [36], while loss-of-function mutants *gi-1* and *gi-2* manifested tolerance to the herbicide tiafenacil through enhanced activity and transcriptional regulation of enzymatic antioxidants such as *APX*, *PrxQ*, *FeSOD3*, *MnSOD*, and *CAT1* [37]. *OsGI* mutant, *osgi-1*, displayed altered sucrose and starch content under natural field conditions [38], while a sorghum *GI* mutant, *sbgi-ems1*, delayed flowering and increased the number of nodes prior to flowering [39]. These diverse findings underscore the intricate role of *GI* in regulating various physiological processes in plants.

### 2.2. Subcellular Localization of GI

Proteins require precise subcellular localization to ensure optimal functionality, as each cellular compartment offers unique environments crucial for effective interactions and functioning. Initial predictions from web-based membrane topology programs indicated that Arabidopsis *GI* encodes a protein with potential membrane-spanning domains [26]. Transient expression of GI-GFP fusion protein in onion epidermal cells demonstrated that GI was localized to the nucleus and formed nuclear bodies [19]. The early flowering 4 (ELF4) interacts with GI within these nuclear bodies to regulate GI’s nuclear compartmentalization [40]. Subsequently, nuclear localization of GI was confirmed in transgenic Arabidopsis overexpressing GI-GFP [24]. Co-localization studies with specific nuclear marker proteins associated with compartments like nucleoli, spliceosomes, heterochromatin beams, and Cajal bodies revealed that GI did not localize to these nuclear compartments, suggesting that GI was not involved in processes such as protein degradation, pre-mRNA splicing, and biogenesis of rRNA and snRNA [40]. The light-dependent formation and distribution of GI nuclear bodies are facilitated by ELF4, which physically interacts with and sequesters GI to inhibit GI’s ability to bind to the promoter of *CONSTANS* (*CO*) [40]. In a related study, early flowering 3 (ELF3) enhances the interaction between GI and constitutive photomorphogenesis 1 (COP1), leading to the formation of nuclear bodies that degrade GI in plants [41].

While GI predominantly resides in the nucleus, it has also been reported to be localized in the cytosol, where it stabilizes the F-box protein zeitlupe (ZTL). The cytonuclear partitioning of core clock components is crucial for proper functioning of the circadian system. Ectopic expression of the N-terminus of ZTL, which houses the light-oxygen-voltage-sensing (LOV) blue-light-absorbing domain targeted by GI, resulted in delayed flowering time and altered hypocotyl length [42]. These phenotypes were attributed to competitive interference of GI’s target, LOV, by the endogenous GI-ZTL complex, suggesting that ZTL regulates the abundance and distribution of GI protein in the cytosol and nucleus. The regulation of GI degradation by COP1 and ELF3, combined with control over its cytonuclear distribution and abundance [42], is crucial for modulating the circadian system and flowering transition in Arabidopsis.

The pivotal role of GI protein in regulating the circadian clock is intricately tied to its subcellular localization. Notably, when CvV:GI-GFP-NLS or CvV:GI-GFP-NES was overexpressed in the *gi-2* mutant background, the GI-GFP fusion protein exhibited preferential localization to the nucleus and cytosol, respectively [35]. Consequently, CvV:GI-GFP-NLS and CvV:GI-GFP-NES differentially complemented flowering regulation in the circadian system: overexpression of CvV:GI-GFP-NLS partially restored early flowering in *gi-2*, whereas CvV:GI-GFP-NES did not exhibit any complementary effect. It is noteworthy that both nuclear and cytosolic GI contribute to regulating the circadian rhythm; however, only the nuclear GI transcriptionally and post-translationally regulates flowering by binding to the promoters of *CO* and *flowering locus t* (*FT*) [43].

## 3. Deciphering the Intricacies of *GI* Transcription and Post-Transcriptional Regulations

Gene expression is pivotal to protein synthesis, and it is tightly regulated by transcription and post-transcriptional mechanisms. Transcription and post-transcriptional mechanisms regulate the amount of mRNA and translation of mRNA into specific proteins, respectively. Despite the pivotal role of transcription and post-transcriptional regulatory processes in cellular functions, detailed exploration of the regulatory mechanisms governing *GI*’s functioning has been limited. *GI* transcript levels are exclusively regulated by the diurnal cycle, with disruptions in circadian clock components leading to alterations in *GI* transcription, which subsequently impacts its functions. Key components of the circadian system, such as the morning-expressed MYB transcription factors *circadian clock-associated 1* (*CCA1*) and *late elongated hypocotyl* (*LHY*), and the evening loop gene, *ELF3*, play crucial roles in transcription regulation of *GI* by binding to its promoter [26,44].

*CCA1* and *LHY* peak in the morning and act as repressors of genes belonging to the evening loop components of the circadian system [45]. Mutations in *CCA1* and *LHY* facilitate earlier transcription of *GI* in the diurnal and circadian cycles, confirming that the repression of *GI* transcription occurs when *CCA1* and *LHY* are expressed in the morning [46,47]. The expressions of *CCA1* and *LHY* were repressed by the *deetiolated1* (*DET1*) [48] and *timing of cab expression 1* (*TOC1*), also known as *pseudo-response regulator 1* (*PRR1*) [49], leading to accumulation of *GI* transcript. The rhythmicity of *GI* transcript levels is disrupted in *elf3* mutant in continuous light (LL) [26], with an increase in the expression of *GI* in *elf3-1*, suggesting that *ELF3* works as a negative regulator of *GI* mRNA abundance [50]. ELF3 exerts rhythmic inhibition of light input pathways around dusk by interacting with COP1 in vivo [41]. Mutations in *COP1* further disrupt the cyclic accumulation pattern of GI [41], underscoring the intricate control exerted by these clock-associated genes on GI’s transcription. The clock proteins light-regulated wd1 (LWD1) and LWD2 are involved in regulating the expression of oscillator genes, *CCA1*, *LHY*, and *TOC1*, and output genes, *GI* and *flavin-binding*, *kelch repeat*, and *f-box1* (*FKF1*) [51], under both SD and LD conditions. The expression of *GI* and other oscillator genes occurs approximately 3 h earlier in the *lwd1lwd2* double mutant [51]. *Time for coffee* (*TIC*) encodes a nucleus-acting protein which is crucial in maintaining the period and amplitude of circadian rhythms [52,53]. The transcript level of *GI* in the *tic* mutants exhibited reduced amplitude and peaked approximately 4 h earlier than in WT plants [52]. The morning genes *PRR9*, *PRR7*, and *PRR5* play partially redundant roles in repressing transcript levels of *GI* [54]. Loss-of-function mutants of *PRR7* and *PRR9* displayed increased *GI* expression at 22 °C, while at 28 °C the repression of *GI* expression by PRR9, PRR7, and PRR5 is limited [54]. 

Recent discoveries highlight the crucial role of histone modifications in the circadian system’s transcriptional network [55]. High expression of osmotically responsive gene 15 (HOS15) is pivotal to the circadian system’s transcriptional network through its interaction with LUX, ELF3, ELF4, and the histone deacetylase 9 (HDA9) in the promoter region of *GI*. This interaction leads to histone deacetylation, resulting in the repression of *GI* expression [56]. The Arabidopsis HDA9 has also been identified as a key player in the regulation of hypocotyl cell elongation and acts by repressing *GI* expression under SD photoperiodic conditions [57]. This dual role of HDA9 underscores its significance not only in the circadian control of *GI* but also in broader physiological processes such as cell elongation.

Following mRNA transcription, myriads of regulatory processes shape gene expression, enabling cells to swiftly modulate protein levels without the need for transcript synthesis or processing [58]. The circadian clock exclusively regulates both the transcript and protein abundance of GI, emphasizing the intricate regulatory layers governing the functioning of *GI*. The protein abundance of GI exhibits a cyclic pattern under varying photoperiods, indicating an additional level of regulation at the post-transcriptional level [27,41]. COP1, an E3 ubiquitin ligase, plays a pivotal role in dark-induced degradation of GI through the 26S proteosome machinery [41,59]. The interaction between COP1 and GI is mediated by ELF3, which acts as a substrate adaptor protein to accelerate GI’s destabilization in the dark [41]. In addition, COP1 facilitates the degradation of GI at low ambient temperature, resulting in delayed flowering independent of the CO pathway [59]. HOS15, characterized as a histone deacetylase, forms a complex with LUX, ELF3, ELF4, and HDA9 to repress transcription of *GI* in the photoperiodic flowering pathway [56]. This association adds another layer to the complex regulatory mechanisms governing GI’s protein abundance. HOS15 participates in the 26S-proteasome-mediated degradation of GI protein, contributing to the coordination of appropriate flowering time responses in the face of changing environmental conditions, including temperature and day length [60]. Furthermore, HOS15 can interact with COP1. Phenotypic analyses of the *hos15cop1* double mutant revealed that repression of flowering by HOS15 is dependent on COP1; however, this complex is attenuated at low ambient temperature, suggesting that HOS15 plays a critical role in low-ambient-temperature-mediated GI degradation independently of COP1 [60].

GI interacts with the LOV domain of ZTL to constitute a complex that co-stabilizes both proteins in the cytosol under blue light [61]. Notably, in *ztl* mutants, GI protein abundance decreases while mRNA levels remain unchanged, suggesting a potential post-transcriptional regulation of GI by ZTL through LOV-mediated heterodimerization [61]. Transgenic Arabidopsis overexpressing the LOV polypeptide exhibits increased GI-HA protein levels, supporting the notion that ZTL-LOV stabilizes GI post-translationally and plays a vital role in the nucleocytoplasmic partitioning of GI [42]. GI’s translational regulation extends beyond the circadian clock, impacting the response to wilt disease in Arabidopsis. Transgenic Arabidopsis overexpressing GI::GI-TAP shows a twofold increase in GI protein levels 24 h after *Fusarium oxysporum* infection compared to the WT, while the *gi-100* mutant displays less severe pathogenic infection than WT [17]. 

The proteasomal degradation of GI was also induced by salt stress. GI interacts with salt overly sensitive 2 (SOS2) under normal conditions and represses SOS2-based activation of SOS1 [20]. On the contrary, high salinity results in the rapid degradation of GI protein in the cytosol, leading to the release of SOS2 kinase to activate the Na^+^/H^+^ antiporter SOS1 by phosphorylation [20,62]. This triggers the export of sodium ions and thus confers salt stress tolerance. Further study reveals that SOS1 directly interacts with GI and plays a specific role in salt compensation of circadian rhythms by stabilizing GI [63]. Moreover, the S-acylation-dependent nuclear import of SOS3 results in the formation of the SOS3-GI-FKF1 protein complex to regulate the transcription of CO under high salinity [64]. The interaction between SOS3 and GI restrained the GI protein to the nucleus, which resulted in the selective stabilization of GI in the cytoplasm to fine-tune the flowering time in a saline environment [64].

## 4. Unraveling the Enigmatic Role of *GI*: A Multifaceted Player in Plant Biology

Despite its discovery over six decades ago, the biochemical function of *GI* has largely remained elusive [33]. While extensive evidence supports the exclusive regulation of GI transcripts and proteins within the circadian clock system, understanding of the molecular-level regulation of GI abundance and its precise biochemical function remains limited [23]. Over the last two decades, *GI* has emerged as a focal point of research, revealing its involvement in a myriad of biological processes in plants. Serving as a pleiotropic gene, *GI*’s diverse roles span various aspects of plant physiology, including circadian clock regulation, light sensing and signaling, flowering time regulation, chlorophyll accumulation, hypocotyl elongation, sugar metabolism, abiotic stress tolerance, and even miRNA processing [36,65].

### 4.1. Stimulus Response

Light is pivotal to shaping key growth and developmental processes in plants, such as photosynthesis, photomorphogenesis, phototropism, and shading escape [66,67]. Sunlight serves as the natural light source and provides the optimal illumination needed for plants to maximize growth and development. Two critical variables, light intensity and spectral quality, significantly impact various aspects of plant physiology. Light intensity affects crucial plant processes such as food production, stem elongation, leaf color, flowering, and overall plant yield [68,69]. Meanwhile, light spectral quality is essential in activating plant photoreceptors, spanning from UV-B to far-red, and includes blue light receptors such as cryptochromes (CRYs), phototropism, and ZTLs [70,71]. Light signaling involves plants’ ability to perceive both the quantity and quality of light through specialized photoreceptors and transduce this information into transcriptional networks that regulate the expression of specific genes to modulate light responses [72]. 

**Figure 1:**
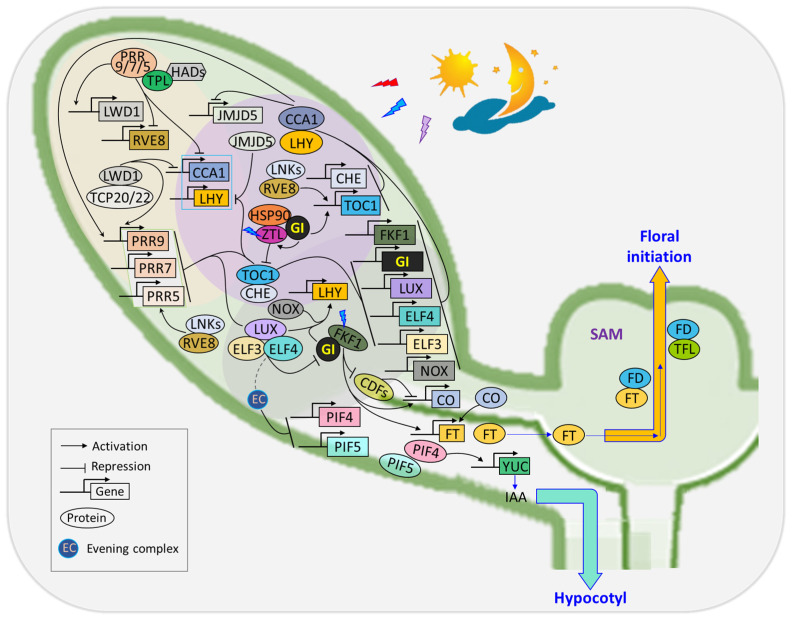
*GI* is implicated in light signaling, contributing to circadian rhythm resetting. In the circadian clock, GI directly activates the expression of *LHY* and *TOC1* to reset the circadian rhythm. GI collaborates with the central clock components in the evening feedback loop, forming a chaperone complex with heat shock protein (HSP90) and ZTL to regulate ZTL stability. This complex promotes the degradation of TOC1, influencing overall clock function. During the evening, TOC1 and evening complex elements reciprocally suppress *GI*. Under long-day conditions, GI interacts with FKF1 to degrade cycling dof factor (CDF), which is a repressor of CO, leading to elevation of *CO* transcript abundance and promotion of *FT* expression to regulate flowering. Additionally, GI integrates light signaling with the circadian clock by regulating PIF proteins (PIF4 and PIF5), which affects output rhythms like hypocotyl elongation.

GI interacts with various proteins at both transcriptional and post-translational levels to influence several biological processes [23,31]. Red and far-red lights are crucial components of light quality and play significant roles in plant growth and development [73]. Phytochromes are central to light signaling pathways primarily via interacting with PHYtochrome-interacting factors (PIFs), which are members of the bHLH transcription factor family and are known to negatively regulate photomorphogenesis in the dark [73,74]. *GI* modulates light signaling by regulating PIF activity and accumulation through multiple mechanisms, including transcriptional and post-translational regulations [75,76]. *GI* influences *PIF4* and *PIF5* mRNA expression during the early night and interacts with PIF7 to repress transcription in response to shade at dusk, illustrating the broader impact of GI on photoperiodic growth and response to environmental cues [77,78]. The interplay between *GI* and light signaling pathways (Figure 1) adds a layer of complexity to our understanding of how plants integrate environmental cues to regulate key physiological processes.

(1) Coordination of red light signaling by *GI*

Phytochromes (Phy) are ubiquitous in land plants and certain algae, serving as serine/threonine kinases that respond differentially to red and far-red lights to orchestrate essential developmental processes. The activation of phytochrome kinase activity by red light and its inactivation by far-red light induces the reversible interconversion between the Pr and Pfr states [79]. In Arabidopsis, five phytochromes (PhyA to PhyE) have been identified, where PhyB to PhyE act as receptors for red light (R, λ = 660 nm), with PhyA serving as the receptor for far-red light (FR, λ = 730 nm). PhyA moderates two distinct photobiological responses: the very-low-fluence response (VLER) and the high-irradiance response (HIR) [80]. 

The responsiveness of *GI* to red light is evident through an increase in *GI* transcript levels in Arabidopsis roots after exposure to red light [81]. *GI*’s regulatory impact on lateral root development under red light involves the modulation of *auxin/indole-3-acetic acid (AUX/IAA)* modules, *LBD16* expression, and the enhancement of *nuleus accumbens-associated protein-1 (NAC1)*, which subsequently promotes *AIR3* expression during lateral root initiation [82]. GI actively participates in light signal transduction by engaging with PhyB and PIFs during hypocotyl elongation [19,75]. Both phyB and gi mutants display elongated hypocotyls in red light [83]. The gi mutants of Arabidopsis exhibit impaired PhyB signaling and elongated hypocotyls relative to the WT plants under saturated red light during seedling de-etiolation. Interestingly, they show little or no responsiveness to continuous far-red light [19], suggesting that GI functions as a positive regulator of PhyB signaling.

(2) Coordination of blue light signaling by *GI*

The integral evening loop components, *ELF3*, *ELF4*, and *ZTL*, intricately regulate light input signals to the circadian clock, influencing plants’ ability to discern varying day lengths [61,84]. The molecular mechanism governing the role of GI in blue light signaling in the circadian oscillator complex revolves around its capability to bind to the blue light receptor ZTL via the light, oxygen, or voltage (LOV) domain, establishing a protein–protein interaction [31]. This interaction post-translationally stabilizes ZTL under blue light conditions, as ZTL possesses an F-box protein [85]. Disruption of the GI-ZTL protein complex results in a significant (four- to fivefold) reduction in peak ZTL levels, emphasizing the crucial role of GI in controlling the circadian period [61]. The HSP90 chaperone plays a pivotal role in carrying GI and facilitating the maturation of ZTL into a vital component of the SCFZTL E3 ligase. This ligase, in turn, targets the central clock protein TOC1 [84] and the morning loop component PRR5 [86] for blue light mediate ubiquitination and subsequent degradation. The late evening phase of GI protein oscillation, coupled with the blue-light-enhanced GI–ZTL interaction, contributes to the establishment and maintenance of an evening-phased post-translational rhythm in ZTL abundance [61]. 

*GI* exhibits dual roles in both photomorphogenic and circadian blue light signaling pathways. Notably, it is differentially required for clock function in constant red versus blue light conditions [87]. Phenotypic analysis of *gi*-mutant alleles under blue light exposure unveils taller hypocotyls compared to the wild type [87]. GI collaborates with blue light receptors, including ZTL, LKP2, and FKF1, to orchestrate the degradation of core clock protein TOC1 and the flowering repressor CDFs. This collaborative effort fine-tunes circadian rhythms and flowering in Arabidopsis [88].

(3) Circadian clock regulation

Plants, constrained by their sessile nature, employ intrinsic regulatory mechanisms such as the circadian clock to navigate and optimize growth and development in response to diurnal environmental cues. The circadian system in Arabidopsis operates on a 24 h cycle, with 16 h of light and 8 h of darkness, providing a commonly adopted temporal framework for organisms to synchronize and coordinate responses to both abiotic and biotic stimuli [89,90]. Functioning as an endogenous time-keeping mechanism, the circadian clock is integral for plants to achieve and sustain fitness, ensuring proper growth and development [91]. The endogenous circadian clock consists of three fundamental modules: (i) an input module that processes information from surrounding environmental cues; (ii) a central oscillator characterized by a negative feedback loop; and (iii) an output pathway acting as a clock-driven module that initiates downstream responses to environmental cues. The central oscillator, a crucial component of the circadian system, encompasses a complex transcription–translation feedback loop (TTFL). This loop, coupled with post-transcriptional and post-translational modifications, regulates various aspects of gene expression, metabolic processes, and physiological adjustments. These mechanisms collectively promote plants’ adaptive responses to diverse environmental conditions [85,92].

The circadian system relies on a multitude of post-transcriptional and post-translational regulatory processes [93]. The morning loop component of the circadian clock comprises MYB transcription factors *CCA1* and *LHY*, along with members of the pseudo-response regulator family, specifically *PRR5*, *PRR7*, and *PRR9* [94]. Upon light signal activation, transcription of *CCA1* and *LHY* is initiated, which in turn promotes the expression of PRR proteins (PRR9, PRR7, and PRR5) in the morning [95]. *PRR7* and *PRR5*, functioning as transcription repressors, provide feedback by suppressing the transcription of *CCA1* and *LHY*. The *reveille clock gene* (*RVE8*) forms an additional feedback loop, which positively regulates PRR5. The protein encoded by PRR5 in turn represses the transcription of *RVE8* [96]. The LWD1 protein is a vital component of the circadian clock that is essential for the expression of *PRR9*, *PRR7*, *PRR5*, and *TOC1* by interacting with their promoters [97] (Figure 1).

The central loop of the circadian clock is comprised of CCA1 and LHY, along with TOC1. This assembly constitutes the negative feedback loop and serves as the oscillator of the circadian clock [98]. Interactions within the negative feedback loop involve transcriptional and post-transcriptional activation and repression processes [94]. The evening loop consists of ELF3, ELF4, LUX, ZTL, and GI [99,100]. CCA1 and LHY proteins peak in the morning and bind to the evening element (EE) on the promoter of TOC1 and other evening-expressed genes to repress their transcription during the day [101,102]. The evening loop connects back to the morning loop through TOC1. As the evening approaches, the abundance of CCA1 and LHY proteins decreases, leading to the accumulation of TOC1, which functions to repress the transcription of CCA1 and LHY [45]. This, in turn, results in the accumulation of GI transcripts and other evening-expressed proteins. Late at night, the GI-ZTL complex promotes the degradation of TOC1 [45], and the evening complex (EC) represses PRR9 and PRR7 transcription, allowing the transcription of CCA1 and LHY to resume at dawn. GI interacts with and regulates key clock components through transcriptional and post-transcriptional processes at specific periods of the day. The expression of GI is regulated by the circadian clock, peaking around 10 h after dawn [26,103]. Circadian regulation of GI, LHY, and CCA1 is altered in gi mutants, underscoring the importance of GI in maintaining circadian amplitude and appropriate period length of these genes [18]. Furthermore, gi mutants displayed disrupted incorporation of light signals into the circadian clock, implicating GI as an active participant in the feedback loop that serves as the central oscillator of the plant circadian system. ZTL achieves stability through its interaction with GI and HSP90 [85]. Other components of the evening loop, ELF3, ELF4, and LUX, function in the evening to repress the morning genes of the PRR family [99] (Figure 1).

### 4.2. Flowering Time Regulation

(1) Orchestrating floral transition in response to photoperiodic signals

The transition from vegetative growth to flowering is a complex process fine-tuned by environmental signals, with the photoperiodic pathway playing a central role. Such transition is influenced by light, photoperiod, and the circadian clock and revolves around key players such as *CO* and the florigen hormone *FT* [104]. Light is perceived by multiple photoreceptors in the leaves, and signal output responses are supervised by the circadian clock. *GI* acts as a gating factor by regulating the *FT* expression in *CO*-dependent and *CO*-independent pathways. *CO*, a pivotal component of the photoperiodic pathway, orchestrates the production of *FT* under long-day photoperiods. Its peak expression in short-day photoperiods is post-dark due to insufficient stabilization by light. CDF1 regulates *CO* transcription by binding to its promoter at sunrise to repress its expression [105]. 

**Figure 2:**
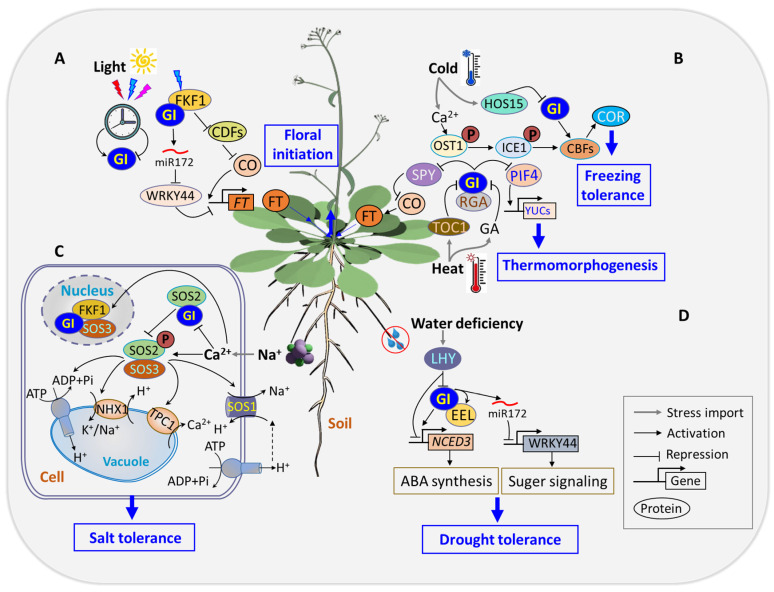
*GI* serves as a central hub protein involved in crosstalk of numerous stress responses and flowering regulation. *GI* functions as a pleiotropic gene that mediates regulatory pathways, influencing various aspects of flowering (**A**) and responses to cold or heat (**B**), salt (**C**), and drought (**D**) stresses. The intricate interplay between these pathways enables *GI* to balance stress responses, promoting both growth and flowering and enhancing plant resilience under adverse conditions.

Flowering time regulation within the circadian clock involves one of the output pathways mediated by GI, which regulates the amount of CO. Under long-day conditions, *GI* and *FKF1* are co-expressed at ZT10 and form a complex. This complex accumulates and peaks in the middle of the day, leading to the degradation of CDF repressors and an increase in *CO* transcript abundance. This consequently promotes the expression of *FT* [21] (Figure 1). Under short-day conditions, where the expression of *GI* precedes that of *FKF1*, the formation of the GI-FKF1 repressor complex is disrupted. This disruption results in a reduction in abundance of *CO* and *FT* [106]. *GI* mutants exhibit reduced *CO* mRNA abundance, further confirming *GI*’s positive regulatory role in flowering time [107]. In addition to the *CO*-dependent regulation of *FT*, *GI* can independently regulate *FT* either by directly binding to its promoter or through microRNA-based regulation. In the latter, GI positively regulates miRNA172 [108], leading to the inhibition of TARGET OF EAT1 (TOE1), TOE2, and TOE3 transcriptional repressors, whose functions are crucial in controlling flowering time [109] (Figure 2A). Additionally, GI inhibits SPY expression in a light-dependent manner. SPY, in turn, suppresses CO and FT expression, with *spy-4* plants mitigating the late-flowering phenotype of *gi-1* plants [110]. In all, *GI* serves as a key mediator between the circadian clock and the master regulators (*CO* and *FT*) in the photoperiodic flowering pathway. The *FT* transcription was activated in leaf vascular tissue (phloem) [111], and its protein was transported to the shoot apex to induce flowering [112].

(2) Stress tolerance

*GI* regulates diverse facets of plant growth and development, from flowering time to circadian clock regulation, light signaling, starch accumulation, chlorophyll biogenesis, and miRNA processing. GI also plays a crucial regulatory role in shaping plants’ response to the environment. Drought is one of the most prominent abiotic stresses that induces a cascade of responses in plants, including the production of reactive oxygen species (ROS), oxidative damage, ion toxicity, and nutrient imbalances [113]. *GI*’s involvement in plant response to drought stress is underscored by its impact on flowering time regulation, which is a crucial mechanism adopted by plants for drought escape [114,115]. Arabidopsis flowering time mutants subjected to conditions triggering drought escape revealed the pivotal roles of *GI*, *FT*, and *TWIN SISTER OF FT* (*TSF*) genes in orchestrating the plant’s response to water availability changes [116]. GI’s involvement in drought escape is elucidated through an ABA-dependent activation of the *SUPPRESSOR OF OVEREXPRESSION OF CONSTANS 1* (*SOC1*). The collaboration between GI and the bZIP transcription factor enhanced em level (EEL) forms a complex that modulates diurnal ABA biosynthesis, which influences drought tolerance [36]. Further insights into *GI*’s role in drought escape unveil a regulatory pathway where GI suppresses *WRKY44* through miRNA172, contributing to the plant’s ability to cope with drought stress [117]. *GI* mutants exhibited abnormal drought escape and tolerant phenotypes, emphasizing the intricate network through which *GI* influences plants’ response to environmental challenges (Figure 2D).

Global climate change has occasioned temperature extremities, posing significant challenges to optimal plant growth, yield, and fruit quality [118,119]. In response to low temperatures, plants activate gene alterations that regulate the production of metabolites, which enhances resistance against damages caused by cold stress [120]. GI plays a crucial role in regulating freeze tolerance, contributing to plants’ ability to withstand cold stress through various regulatory mechanisms. Transcriptome profiling of Arabidopsis exposed to cold stress revealed an upregulation of *GI* transcripts, facilitating cold stress acclimation independently of C-repeat binding protein (CBP) [121]. Under constitutive cold stress, GI is induced, and the *gi-3* mutant exhibits decreased cold tolerance and impaired acclimation compared to the WT [122]. This emphasizes the significance of GI in plants’ adaptive response to cold stress conditions. HOS15, a transcriptional repressor of GI, operates independently of COP1 in mediating GI’s degradation and regulation of flowering time in response to low ambient temperature [60]. The interaction between GI and the CDF module plays a pivotal role in mediating the transcriptional regulation of CDFs, thereby influencing freezing tolerance in Arabidopsis [76]. This intricate module adds another layer to the regulatory network through which *GI* contributes to plants’ ability to tolerate and acclimate to cold stress (Figure 2B)**.**

Furthermore, temperature signals integrate into the clock transcriptional circuitry through the EC consisting of ELF3/4 and LUX. This regulation extends to the transcription of *PRR9*/*PRR7*, *GI*, *LUX*, and *PIF4* in response to both temperature changes and variations in steady-state growth temperature [123]. Under long-day conditions, elevated temperatures promote the accumulation of GI protein. GI then interacts with and stabilizes the repressor of ga1-3 (RGA), a DELLA protein, which consequently dampens PIF4-mediated thermomorphogenesis [124]. Conversely, under short days with reduced GI accumulation, RGA undergoes rapid degradation through the gibberellic-acid-mediated ubiquitination–proteasome pathway to facilitate thermomorphogenic growth [124]. These findings suggest that the GI–RGA–PIF4 signaling module facilitates day-length-dependent plant thermomorphogenic responses (Figure 2B). 

The salt overly sensitive (SOS) pathway is a crucial mechanism employed by plants to manage salinity stress. The SOS pathway ensures the exclusion of excess sodium ions from cells and maintains Na^+^/K^+^ homeostasis [125]. This pathway involves the induction of SOS pathway genes under salinity stress conditions [126]. The SOS pathway comprises three core components: SOS3 (a Ca^2+^ of the calcineurin B-like, CBL, family), SOS2 (a CBL-interacting protein kinase, CIPK), and the Na^+^/H^+^ antiporter, SOS1/NHX7 [127]. The plasma-membrane-localized SOS1, acting as a sodium exporter, plays a crucial role in excluding sodium from the root [62]. Sodium that is taken up into plants through nonselective cation channels (NSCCs) can be partly returned to the soil by the action of SOS1/NHX7 [128]. Other vacuolar localized NHX proteins like NHX1 and NHX2 are responsible for sodium sequestration and potassium homeostasis in the vacuole [129]. Sodium absorption by plant cells in the roots, followed by transfer to the shoot, can lead to cellular damage. To reduce shoot Na^+^ levels, Na^+^ can be sequestered in the vacuole through NHX1 and NHX2. This process, in turn, increases vacuolar pH and prevents cell expansion [129]. Additionally, sodium can be loaded into the phloem by HKT1 for transportation back to the root [128]. GI emerges as a key regulator (Figure 2C) of salt stress response by interacting with essential components of the SOS signaling pathway [20]. Under normal growth conditions, GI competitively binds to SOS2 kinase to inhibit its phosphorylation-dependent activation of SOS1. This interaction prevents the efflux of Na^+^ ions. However, under salt stress, GI degradation by the 26S proteasome releases SOS2 to form an active SOS2/SOS3 protein kinase complex, leading to the activation of the Na^+^/H^+^ antiporter SOS1. 

The degradation of GI results in the export of Na^+^ ions out of the plasma membrane, establishing salt stress tolerance. Transgenic plants overexpressing GI exhibit hypersensitivity to salt stress by sequestering SOS2, emphasizing the intricate role of GI in salt stress response [20]. Under salt stress conditions, S-acylated and nuclear-localized SOS3/CALCINEURIN B-LIKE4 stabilize GI in the nucleus and recruits FKF1 to form a GI-FKF1-SOS3 complex. This complex actively participates in sustaining the transcription of *CO* and *FT* and adjusts flowering time in response to saline environments [64]. In contrast, GI degradation in the cytosol releases SOS2, contributing to salt stress tolerance [64]. Overexpression of GIGANTEA-like PagGIs in wild-type Arabidopsis induces early flowering and sensitivity to salt stress. Furthermore, the overexpressing PapGIs in the *gi-2* mutant partially or completely restore its delayed flowering phenotype and confer salt stress tolerance [130].

*GI* exhibits a dual role in oxidative stress response, showcasing its intricate involvement in the delicate balance between tolerance and susceptibility. Studies on Arabidopsis *gi-3* mutants reveal enhanced tolerance to oxidative stress, attributed to the activation of key antioxidant genes and subsequent elevation in the activities of superoxide dismutase (SOD) and ascorbate peroxidase (APX) enzymes [131]. Paradoxically, *GI* demonstrates a negative regulatory role in resistance to the herbicide butafenacil, which is known to induce oxidative stress by disrupting protoporphyrinogen IX oxidase activity. The *gi-1* and *gi-2* mutants exhibit resistance to butafenacil, highlighting the complex role of GI in oxidative stress response [132]. Furthermore, loss-of-function mutations in *GI* confer resistance to the PPO-inhibiting herbicide tiafenacil, revealing a transcriptional regulatory mechanism that links *GI* to oxidative stress responses [37]. The *gi-1* and *gi-2* mutants exhibit robust resistance, with survival rates of 97% and 83%, respectively, compared to 56% in WT and *GI*-overexpression lines. Additionally, *gi-1* and *gi-2* mutants showed increased transcriptional expression and enzyme activity of antioxidants, emphasizing the role of *GI* in modulating oxidative stress responses [37]. Another *GI* mutant allele, *gi-3*, displays resistance to oxidative agents such as paraquat and hydrogen peroxide [131]. 

### 4.3. Chlorophyll Accumulation Is Regulated by GI in Plants

Plants exhibit the capacity to regulate chlorophyll distribution across tissues, which balances their visibility and functionality. In petals, chlorophyll accumulation is limited to preserve the conspicuousness of flowers, while leaves accumulate substantial amounts crucial for photosynthesis [133]. Chloroplasts house the chlorophyll, which serves as sites for light energy capture and conversion during photosynthesis [134]. *GI*, modulated by the circadian clock, plays a pivotal role in chloroplast biogenesis in Arabidopsis. The *gi-2* mutant displays reduced sensitivity to the chloroplast biogenesis inhibitor lincomycin, maintaining higher photosynthetic protein levels. Conversely, wild-type and *GI*-overexpressing transgenic lines exhibit lincomycin hypersensitivity, leading to variegated leaves and reduced photosynthetic protein abundance [132]. This underscores *GI*’s involvement in chloroplast and chlorophyll biogenesis. In a related study, Arabidopsis *GI* mutant alleles (gi-3, gi-4, gi-5, and gi-6) show significantly higher seedling chlorophyll content than the wild type after paraquat treatment, confirming *GI* as a negative regulator of chlorophyll biogenesis [135].

Nitric oxide treatment suppresses *GI* mRNA abundance, resulting in increased chlorophyll content in Arabidopsis [38]. Under the long-day photoperiod, *gi-201* and *gi-2* mutants maintain green leaves for 32 days post-emergence compared to the wild type. Conversely, gi-2 mutants overexpressing GI exhibit leaf tip yellowing at 24 days post-emergence, which intensified at 28 days post-emergence. Chlorophyll fluorescence (Fv/Fm) was higher in *gi-201* and *gi-2* mutants than in the wild type, highlighting *GI*’s dual role as a positive regulator of leaf senescence and a negative regulator of chlorophyll accumulation [35]. Mutation in *GI* impacts the *CAB2* gene, which is a key component of the light-harvesting complex of photosystem II [18]. Silencing of GI’s paralog *PhGI1* in *Petunia hybrida* results in *phgi1* with greener apical regions and increased chlorophyll accumulation compared to the wild type [135]. Conversely, loss of function of a rice *GI* mutant, *osgi*, displayed significantly reduced leaf chlorophyll content compared to the wild type, indicating species-specific variations in *GI*-mediated chlorophyll regulation [29]. 

### 4.4. GI Regulates Stomatal Opening in Plants

Stomata are minute pores on the epidermis of leaves and stems which connect internal air spaces with the external atmosphere. Guard cells control the stomatal aperture, modulating gas exchange and water loss through transpiration in response to environmental and exogenous signals [136,137]. Blue light acts as a stomatal opening signal, perceived by receptor kinases phot1 and phot2, which activates the plasma membrane H^+^-ATPase [138,139]. GI functions in the blue light signaling pathway by directly binding to the LOV motif of ZTL, LKP2, and FKF1 [21]. Core components of the photoperiodic flowering pathway, including *cryptochromes* (*CRY*), *GI*, *CO*, *EFL3*, *FT*, *TSF*, and *suppressor of overexpression of co1* (*SOC1*), are expressed in guard cells to regulate light-induced stomatal opening [140,141]. Overexpression of these components leads to opened stomata, while knockout mutants exhibit reduced light-induced stomatal opening. *CRYs* are blue light photoreceptors which promote floral transition [142] by preventing ubiquitination of GI and CO proteins [41,143], thereby controlling stomatal opening through FT and TSF in response to photoperiod [144]. TSF mutants and overexpression in phot1 and phot2 mutants display suppressed and constitutive stomatal opening, respectively [144]. Similarly, *gi-1* and *co-1* mutants exhibit suppressed blue-light-induced stomatal opening, while GI and CO overexpression results in constitutive open-stomata phenotypes. The GI- EEL complex binds to the promoter of 9-cis-epoxycarotenoid dioxygenase 3 (NCED3) and activates its transcription to mediate stomatal opening. The *gi-1*, *eel*, and *gi-1eel* mutants, compared to the wild type, are hypersensitive to drought stress due to uncontrolled water loss from increased stomatal aperture [36].

### 4.5. GI’s Role in Plant Sugar Signaling

In plants, naturally occurring sugars, including sucrose, fructose, glucose, trehalose, and their derivatives such as pectin, cellulose, hemicellulose, callose, and starch, play a dual role as both the primary source of energy for cellular metabolism and the structural components of plant cells [145]. These sugars also act as signaling molecules within the circadian clock system to regulate various aspects of cellular development, such as floral transition [146], hormonal pathway signaling [147], and innate immunity [148]. Plant cells employ signaling mechanisms to perceive carbon and energy status and dictate metabolic adjustments. Under carbon limitation, SnRK1 activity prolongs the circadian period, while sucrose shortens it through the T6P-SnRK1 complex acting on the clock oscillator gene PRR7 [149,150]. The circadian clock slows down in the dark due to the absence of light and cellular metabolism but can be sustained by the addition of sugar. Sucrose sustains the circadian rhythm in the dark by stabilizing GI protein through a regulatory mechanism dependent on the F-box protein ZTL and constitutive response1 (CTR1), a negative regulator of ethylene signaling [151]. The regulation of *GI* expression by sucrose suggests a connection that measures and reports metabolic status to alter or reset the circadian clock [152].

Eimert [153] reported an increase in sugar accumulation in the WT in response to cold treatment, while the *gi-3* mutant displayed a significant reduction in soluble sugar content, attributing the sensitivity of the *gi-3* mutant to cold treatment to the constitutive reduction in soluble sugars. This affirms that *GI* has a direct connection with sugar metabolism. In contrast, rice plants carrying a null mutation in the rice homology *OsGI* (*osgi-1*) recorded higher leaf sucrose and starch at most points in time under natural field conditions [29]. Similarly, monogenic recessive mutants *gi-1*, 2, and 3 caused an increase in both late flowering initiation and increased starch content in Arabidopsis [154]. GI interacts with trehalose-6-phosphate synthase 8 (TPS8) to form a complex which may have a direct influence on carbohydrate metabolism [155]. Under LDs, drought stress induces the expression of *GI*. Modes of GI-dependent but CO-independent pathways include the activation of *miR172*, thus inhibiting the transcription of *WRKY44* [117], which was considered to be involved in sugar metabolism and signaling, indicating a role of *GI*–*miRNA172* in drought escape and defense by affecting sugar signaling (Figure 2D)**.**

### 4.6. GI’s Unexplored Role in Anthocyanin Metabolism

Anthocyanins are a class of polyphenolic pigments in plants, which are induced and accumulate in response to various environmental signals, with their biosynthesis regulated by transcription factors. Environmental cues such as light, low temperature, drought, and salinity significantly influence anthocyanin biosynthesis [156,157]. Among these cues, light stands out as the most prominent regulatory factor in the anthocyanin biosynthesis pathway. While *GI* has been primarily linked to stress response regulation in plants, its involvement in anthocyanin metabolism has received limited attention. A study by Odgerel [158] has shed light on a novel role of *GI* in anthocyanin metabolism in potatoes. The research revealed that mutants with repressed *StGI.04*—specifically aG153, aG144, and aG152 tuber peels—exhibited a 52%, 36%, and 31% reduction in anthocyanin content, respectively, compared to the wild type (DES). This discovery highlights the unexplored connection between *GI* and anthocyanin regulation, opening avenues for further investigation into this intriguing aspect of plant physiology.

### 4.7. Integrative Role of GI in Hormonal Signaling

Phytohormones play pivotal roles in orchestrating plant growth and development, serving as key regulators that enable plants to respond systematically to environmental changes [159]. The circadian clock actively participates in hormonal signaling pathways, exerting regulatory control over components of auxin, jasmonate, brassinosteroids, cytokinin, GA, and abscisic acid [160]. These hormones, in turn, reciprocally influence the circadian clock system, establishing a feedback loop that fine-tunes the oscillator’s activity [161]. *GI* has emerged as a central player in integrating hormonal signals to regulate diverse processes in plants. This interplay between the circadian clock and hormonal regulation underscores the intricate web of molecular interactions that govern plant growth, emphasizing the multifaceted role of *GI* in these dynamic processes.

(1) The role of *GI* in ABA-mediated responses to drought stress

ABA stands out as the most extensively studied signal governing gene expression in response to drought stress perception. Drought induces accumulation of the stress hormone ABA and activates its downstream signaling pathway, which takes charge of promoting stomatal closure to reduce the transpiration rate. Notably, ABA orchestrates gene expression in a meticulously organized diurnal cycle, ensuring that the physiological traits under ABA regulation manifest at specific time periods. *GI*, a key regulator of photoperiod-dependent flowering and the circadian rhythm, emerges as a central player in this intricate ABA regulatory network, acting as a key gatekeeper for ABA-regulated transcriptional and physiological responses [162]. The modulation of *GI* signaling by ABA contributes to the transcriptional upregulation of *FT*, *TSF* and *SOC1*, which ultimately promotes drought escape in Arabidopsis [163]. 

Moreover, *GI* plays a vital role in regulating the synthesis and signaling of ABA. GI interacts with EEL, a basic Leu zipper (bZIP) transcription factor involved in ABA-regulated gene expression during seed dehydration [36]. This heterodimer complex promotes ABA biosynthesis by directly activating the diurnal expression of *NCED3*, a rate-limiting enzyme in ABA biosynthesis in plastids, to enhance drought tolerance in Arabidopsis [36] (Figure 2D). The endogenous ABA in turn promotes flowering via upregulating the expression of *FT* to avoid prolonged exposure to drought [116]. This highlights the molecular crosstalk between the circadian clock and ABA signaling to cope with drought.

(2) *GI*’s involvement in gibberellin signaling for hypocotyl elongation

Gibberellins (GAs) is a crucial phytohormone, pivotal for promoting cell elongation, facilitating the overall growth of plants. GA signaling is strongly linked with the circadian clock in the regulation of developmental processes. On the one hand, GAs operate as an output module within the circadian network to affect the function of the circadian clock. On the other hand, the clock directly governing the diurnal accumulation of GA levels by regulating the transcription of genes, which involved in the biosynthesis and catabolism of GAs. Moreover, the clock governs the responsiveness to GAs by controlling the expression of the GA receptor gene ga insensitive dwarf 1 (GID1) [164]. GI’s regulatory role in GA signaling hinges on its ability to stabilize DELLA proteins, including repressor of ga1-3 (RGA), gibberellic acid insensitive (GAI), and rga-like protein 3 (RGL3). These DELLA proteins function as negative components within the GA signaling pathway, allowing GI to precisely modulate the timing of GA sensitivity [165]. GI interacts with and stabilizes RGA in the context of their GA-mediated degradation and plays a vital role in the circadian gating of GA signaling [165]. A recent report revealed that DELLA is mono-O-fucosylated by the spindly (SPY), a novel O-fucosyltransferase, thereby activating DELLA by promoting its interaction with key regulators like PIF3 and PIF4 [166]. GI interacts with SPY, a negative regulator of gibberellin signaling, to regulate hypocotyl elongation [110]. However, it is unclear whether the GI–SPY interaction has any implications for DELLA O-fucosylation. GI interacts with PIFs and modulates their stability and activity [75], which in turn regulates the expression of phytohormones biosynthesis and signaling. PIF1/PIL5 represses *gibberellin 3*-*oxidase* 1 (*GA3ox1*) and *GA3ox2*, which encode enzymes to produce GA1 and GA4 [167]. *PIF1*/*PIL5* actives the expression of *GAI* and *RGA* by directly binding to their promoters [168]. Furthermore, *GI* also regulates the transcription levels of *CCA1* and *LHY*, which are involved in circadian clock regulation and responsiveness to GAs [75].

(3) *GI*’s impact on phytohormones in biotic stress response

Salicylic acid (SA) is an important phytohormone best known for regulating plant responses to pathogen infections. Jasmonates (JA) are phospholipid-derived phytohormones that mediate both developmental processes and responses to environmental stresses. *GI*, beyond its role in the circadian clock, has been identified as a regulator which influences plant responses to biotic stress through the modulation of phytohormones such as salicylic acid (SA) and jasmonates (JA). Recent research reveals that *GI* expression promotes disease severity by downregulating the SA accumulation and altering the phenylpropanoid pathway in both Arabidopsis and wheat during *Bipolaris sorokiniana* infection. This downregulation contributes to the suppression of pathogenesis-related responses, ultimately rendering the plants susceptible to the disease [169]. It seems that the *GI* gene acts as a negative regulator in the SA signaling pathway, and the downregulation of *GI* could be beneficial in generating disease tolerance. In *Arabidopsis thaliana*, *GI* has been shown to downregulate JA signaling as well, leading to reduced severity of spread and damage caused by pathogenic infections. The *gi-100* mutant, exhibiting late flowering, demonstrated heightened susceptibility to *Hyaloperonospora arabidopsidis* infection, with the regulatory involvement of *phytoalexin deficient 4* (*PAD4*) in the pathogen infection phenotype [82]. The *gi-100* mutant displayed enhanced tolerance to wilt disease, showcasing a positive correlation between late flowering and resistance to *Fusarium oxysporum* [22,170]. The relative transcript expression of *coronatine insensitive 1* (*COI1*) and *plant defensin 1.2* (*PDF1.2*), marker genes of the JA pathway, is significantly upregulated in the *gi-100* mutants compared to Col-0 plants, while the *isochorismate synthase 1* (*ICS1*) and *nonexpressor of pathogenesis-related genes 1* (*NPR1*), markers of the SA pathway, are downregulated [17]. These results suggest that the *GI* module promotes susceptibility to F. oxysporum infection by inducing the SA pathway and inhibiting JA signaling in Arabidopsis. These findings underscore *GI*’s intricate role in plant defense mechanisms, shedding light on its impact on phytohormonal regulation during biotic stress responses.

(4) *GI*’s role in brassinosteroid signaling pathway

Brassinosteroids (BRs) are essential steroid hormones that play pivotal roles in plant signaling, contributing to cell expansion, cell division, and crucial developmental processes such as etiolation and reproduction [171]. Loss-of-function mutants of GI (abz126) displayed altered responses to specific compounds: insensitivity to paclobutrazol- (PAC), abnormal reactions to benzylaminopurine (BAP), and insensitivity to brassinolide (BL) [172]. The observed phenotypic variations in *GI* mutants suggest a direct association between the loss of function of the GI gene and disruptions in brassinosteroid signaling. UBP12/UBP13 are two novel positive regulators of BR signaling that can remove K-48- and K-63-linked ubiquitin from pBES1/BES1, rescuing them from destruction [173]. UBP12 and UBP13 interact with deubiquitinate BES1 to stabilize its protein, which acts as a positive regulator in BR signaling [173]. In addition, UBP12 and UBP13 act as components of the ZTL-GI photoreceptor complex to stabilize GI, ZTL, and TOC1 [174]. *GI*’s regulatory role in the brassinosteroid pathway underscores its significance in coordinating plant responses to hormonal signals, influencing aspects of growth, flowering, and sensitivity to specific compounds. These findings highlight *GI* as a key player in the intricate network of brassinosteroid signaling, contributing to the modulation of plant development and responses to external stimuli.

## 5. Conclusions

*GI* stands out as a crucial and evolutionary conserved nuclear protein, tracing its roots back to ancient origins. It plays a central role in orchestrating a complex clock-associated feedback loop, influencing a myriad of processes that govern diverse aspects of plant growth, development, and responses to environmental stresses. Despite its recognized multifunctionality, the specific functional domains of GI and their roles remain shrouded in mystery, posing a challenge due to its substantial size and involvement in various pathways.

The exploration of *GI*’s roles in flowering time regulation, circadian clock control, and light signaling is ongoing, revealing lesser-known functions like sucrose signaling, chlorophyll accumulation, and oxidative stress resistance. Recent revelations, including *GI*’s participation in salt and drought tolerance, emphasize that our understanding of its diverse functions is far from complete. Moreover, GI interacts with proteins associated with circadian rhythm, flowering time, and stress response, and various signaling pathways highlight its multifaceted nature. These interactions, occurring at multiple levels and showcasing conservation across the entire plant kingdom, underscore its significance in determining crop harvests and vegetation times.

In conclusion, the intricate functions of *GI* in plant biology, spanning developmental processes and stress responses, necessitate continuous exploration. The ongoing research, uncovering new roles such as salt and drought tolerance, indicates that our knowledge of this essential plant protein is still evolving. The pursuit of understanding *GI*’s roles in flowering time regulation, circadian clock control, light signaling, and emerging functions like salt tolerance indicates an exciting future for *GI* investigations. The existence of paralogues in different plant species and structural variations in gene composition add complexity to the study of *GI* function, which necessitates further exploration. In summary, *GI* emerges as a key regulator with diverse functions, significantly contributing to plant adaptability in dynamic environments.

## Data Availability

No new data were created or analyzed in this study. Data sharing is not applicable to this article.
